# Effects of supplemental chromium picolinate and chromium nanoparticles on performance and antibody titers of infectious bronchitis and avian influenza of broiler chickens under heat stress condition 

**Published:** 2017-09-15

**Authors:** Farhad Hajializadeh, Hasan Ghahri, Alireza Talebi

**Affiliations:** 1 Graduate Student, College of Veterinary Medicine, Urmia Branch, Islamic Azad University, Urmia, Iran;; 2 Department of Animal Science, College of Veterinary Medicine, Urmia Branch, Islamic Azad University, Urmia, Iran;; 3 Department of Poultry Health and Diseases, Faculty of Veterinary Medicine, Urmia University, Urmia, Iran.

**Keywords:** Avian influenza, Chromium, Heat stress, Infectious bronchitis, Nano-chromium

## Abstract

This experiment was carried out to investigate the effects of different levels chromium picolinate (CrPic) and chromium nanoparticles (nano-Cr) on the performance and immune function of broilers under heat stress condition. A total of 320 Ross 308 broiler chicks (from 21 to 42 days) were assigned randomly into eight treatment groups (four replicates per treatment, and 10 chicks per replicate) and be reared at either thermoneutral (21 ˚C) or heat stress (36 ^◦^C). The treatments were control (T1) group without supplementation and heat stress, T2 as a heat stress group without supplementation, T3, T4 and T5 groups which were supplemented with 500, 1000 and 1500 ppb CrPic in diet with heat stress, respectively and T6, T7 and T8 groups which were supplemented with 500, 1000 and 1500 ppb nano-chromium in diet under heat stress, respectively. Supplementation of chromium and nano-chromium improved performance including weight gain and feed conversion ratio of heat-stressed chickens. Antibody titers against avian influenza (AI) and infectious bronchitis (IB) at 21 to 42 days of age in broilers fed supplemental chromium and nano-chromium were higher than broiler chickens fed control diet (*p *< 0.05). Nano-chromium supplementation at level of 1000 ppb and CrPic at level of 1500 ppb improved the antibody titers against AI and IB of broilers under heat stress conditions. It can be concluded from these findings that dietary supplementation of CrPic and nano-Cr can improve performance and antibody titers against AI and IB under heat stress conditions in broilers.

## Introduction

Heat stress is one of the most important environmental stressors challenging poultry production, which causes economic losses in poultry industry worldwide. Supplementation of organic chromium (Cr) can improve performance of broiler chickens.^1^ It has been reported that chromium nanoparticles (nano-Cr) in the rat diet can significantly increase average body weight gain, feed efficiency and chromium content of the organs, reduce body fat ratio and serum insulin concentration.^2^ Chromium was suggested to be a bioactive part of a biomolecule called chromodulin, which is a part of insulin signaling pathway and appears to affect carbohydrate and lipid metabolism via the action of insulin.^3^

 Heat stress has immunosuppressive effects reducing systemic humoral immune responses.^4^^-^^6^ Chromium picolinate (CrPic) is an organic source of chromium that has high bioavailability.^7^^,^^8^ The primary role of Cr in metabolism is to potentiate the action of insulin through its presence in an organometallic molecule called glucose tolerance factor (GTF).^9^ Immunological functions enhance by trivalent Cr and its effects seem to be more pronounced during the times of stress.^10^ Also, occurrence of reduced bursa weight in broilers subjected to heat stress as well as decreased numbers of lymphocytes in the cortex and medulla areas of the bursa in these animals have been demonstrated previously.^11^ Organic Cr supplementation has shown be effective in diminishing adverse effects of heat stress in broilers.^12^^,^^13^ It has been observed that broilers under heat stress condition have lower levels of total circulating antibodies as well as lower specific IgM and IgG levels during primary and secondary humoral responses and reduced thymus, bursa, spleen and liver weights.^14^ Supplementation of chromium methionine (CrMet) at level of 800 ppb can improve immune responses to Newcastle disease and infectious bronchitis (IB) of broiler under heat stress condition.^15^ It has been reported that antibody production in young broiler chickens decreases in heat stress condition^16^ and antibody titer against IB improves in broiler chicks fed 400 ppb of CrPic.^17^ This study was conducted to investigate the effects of CrPic and nano-Cr on performance and antibody titers against AI and IB under heat stress condition in broiler chickens.

## Materials and Methods

A total of 320 day-old Ross 308 broiler chickens fed with corn-soybean meal based diet as shown in Table 1 and were allocated into 8 treatments in a completely randomized design at 21 days of old. Treatment 1 (control) received standard control diet without heat stress.^18^ Treatment 2 was heat stress group without supplementation. In treatments 3-5 chromium at 500, 1000 and 1500 ppb in the form of CrPic (Sigma-Aldrich, St. Louis, USA) was added at rates of 418.30, 836.60 and 1254.90 mg per 100 kg of diet under heat stress, respectively. In treatments 6, 7 and 8 nano-Cr at 500, 1000 and 1500 ppb of diet was added at rates of 418.30, 836.60 and 1254.90 mg per 100 kg under heat stress, respectively. Chickens raised under heat stress condition were maintained under environmental temperatures of 36 ± 1 ˚C from 21 to 42 days of age (from 10:00 to 18:00 hr).

**Vaccination program. **Chickens were vaccinated against Newcastle disease (ND), IB and AI. Vaccination against ND and IB were performed using live vaccines including bivalent ND + IB (Cevac® Vitabron; Ceva Santé Animale, Libourne, France) at day one via eye drop method, mono-valent B1 (Pestikal B1 SPF; Genera, Rakov Potok, Croatia) by eye drop at day 9 and monovalent H120 (Bioral H120; Merial, Lyon, France) at day 15 by drinking water method as well as a killed vaccine against ND and AI (Gallimune 204; Merial) at day 9 via intra-muscular injection.

**Nano-chromium preparation. **Chromium nanoparticle was processed by a wet polish method in a dry cryo-nanonization grinding system integrated with a size separator (Hsin Fang Nanotechnology, Tainan, Taiwan). Briefly, 10 g raw CrPic material was added to 250 mL of 95% ethanol. Then, passed through appropriate sized end-plates sieves to collect nano- and micro sized particles of Cr.

**Fig. 1:**
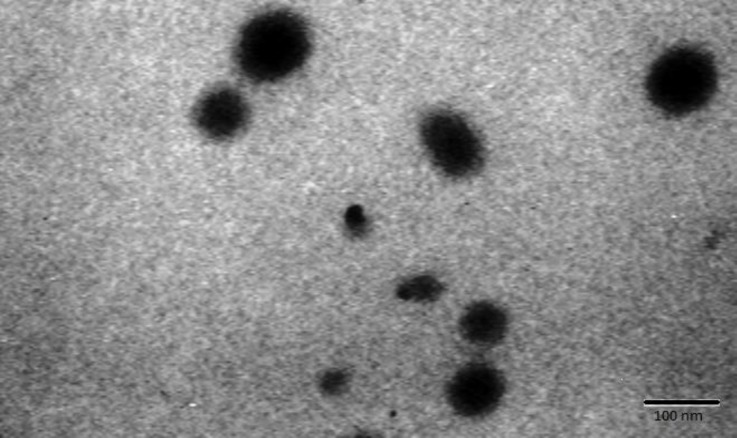
Transmission electron microscopy (TEM) image of chromium nanoparticles showing dense round electron dense bodies (Bar = 100 nm

**Nanoparticle size determination. **A small amount of nano-Cr powder was added to 5.00 mL of methanol and shaken in a water bath for 5 min. Then, small drop of the sample was added to a 4 mm diameter sample and 100 mesh formvar coated copper grid (TAAB Laboratories Equipment Ltd., Aldermaston, UK). Nanoparticles were examined under transmission electron microscope at 75 KV and electron micrographs were taken. The particle size was determined by a transmission electronic microscope (Bio Twin 100; Philips, Eindhoven, The Netherlands) and is shown in Figure 1.

**Performance evaluation. **Feed intake and body weight gain were recorded weekly (21, 28, 35 and 42 days of age) during the whole experimental procedure and data were analyzed for growth period (21-42 days of age). Also, protein efficiency ratio and European performance efficiency factors were analyzed for whole experimental period.

**Sampling.** At 21, 28, 35 and 42 days of age, blood samples were collected from the wing vein of birds (eight chicks per treatment).

**Hemagglutination inhibition test. **Serum obtained by centrifugation of samples and subjected to hemagglutination inhibition test (HI) according to OIE protocol.^19^ Briefly, after two-fold serial dilutions of sera, 4HA AI (H9N2) and same volume (25 µL) of diluted sera were added in each well of 96 well microplate. After 45 min incubation at room temperature, 25 µL of 1% chicken red blood cell (RBC) was added and after 30 min incubation at room temperature, the last well which had a complete inhibition was considered as antibody titer.

**Table 1.** Ingredient and nutrient composition of diet in 21- 42 days of age

**Items**	**Groups**							
	**Control**	**Heat stress**	**500 ppb CrPic**	**1000 ppb CrPic**	**1500 ppb CrPic**	**500 ppb Nano-CrPic**	**1000 ppb Nano-CrPic**	**1500 ppb Nano-CrPic**
***Ingredient (%)***								
**Corn**	69.00	69.00	69.00	69.00	69.00	69.00	69.00	69.00
**Soybean meal (44% CP)**	26.00	26.00	25.99	25.99	24.88	25.99	25.99	24.88
**Corn gluten meal**	3.00	3.00	3.00	3.00	3.00	3.00	3.00	3.00
**Soybean oil**	2.55	2.55	2.55	2.55	2.55	2.55	2.55	2.55
**Dicalcium phosphate**	1.70	1.70	1.70	1.70	1.70	1.70	1.70	1.70
**Calcium carbonate**	1.20	1.20	1.20	1.20	1.20	1.20	1.20	1.20
**Salt**	0.30	0.30	0.30	0.30	0.30	0.30	0.30	0.30
**DL-Methionine**	0.10	0.10	0.10	0.10	0.10	0.10	0.10	0.10
**L-Lysine HCl**	0.15	0.15	0.15	0.15	0.15	0.15	0.15	0.15
**Vitamin premix** a	0.25	0.25	0.25	0.25	0.25	0.25	0.25	0.25
**Mineral premix** b	0.25	0.25	0.25	0.25	0.25	0.25	0.25	0.25
**CrPic mg per 100 kg**	-	-	418.30	836.60	1254.90	-	-	-
**Nano-CrPic mg per 100 kg**	-	-	-	-	-	418.30	836.60	1254.90
**ME, kcal kg** ^-1^	3200	3200	3200	3200	3200	3200	3200	3200
**Crude protein (%)**	19.00	19.00	19.00	19.00	19.00	19.00	19.00	19.00
**Methionine (%)**	0.40	0.40	0.40	0.40	0.40	0.40	0.40	0.40
**Lysine (%)**	0.95	0.95	0.95	0.95	0.95	0.95	0.95	0.95
**Calcium (%)**	0.90	0.90	0.90	0.90	0.90	0.90	0.90	0.90
**Available phosphorus (%)**	0.40	0.40	0.40	0.40	0.40	0.40	0.40	0.40

a Vitamin premix provided the following per kilogram of diet: Vit. A, 8,250.00 IU; Vit. D3, 1,000.00 IU; Vit. E, 11.00 IU; Vit. B12, 0.01 mg; Vit. K, 1.10 mg; niacin, 53.00 mg; choline, 1,020.00 mg; folic acid, 0.75 mg; biotin, 0.25 mg and riboflavin, 5.50 mg.

b Mineral premix provided the following per kilogram of diet: Mn, 55.00 mg; Zn, 50.00 mg; Fe, 80.00 mg; Cu, 5.00 mg; Se, 0.10 mg; I, 0.36 mg and Na, 1.60 g.

**Enzyme linked immunosorbent assay (ELISA). **Antibody titers against IB disease virus were determined by ELISA kit (Idexx Inc., Westbrook, USA) according to the manufacturer’s instructions.

**Statistical analyses. **Data were analyzed by General Linear Models using the PROC GLM procedure of SAS (version 9.2; SAS Institute, Cary, USA). Significant differences (*p *< 0.05) among treatment means were determined using Duncan new multiple range test. 

## Results

Effects of Cr-Pic and nano-Cr on performance of broiler chickens are shown in Table 2. Heat-stressed group showed lower body weight and higher feed conversion ratio compared to control group (*p *< 0.0001). 

**Table 2:** Effect of chromium picolinate and chromium nanoparticles supplementation on performance during 21-42 days of age. Data are presented as mean ± SEM

**Groups**	**Body weight at 42 day (g)**	**Body weight gain (g)**	**Feed intake (g)**	**FCR** 1	**EPE** 2	**PI** 3	**PER** 4
**Control**	2347.50^a^	1822.00^a^	3309.40^a^	1.82^d^	295.70^a^	55.08^a^	2.90^a^
**Heat stress**	1904.50^e^	1264.50^f^	2890.90^c^	2.35^a^	171.00^f^	39.30^d^	2.06^d^
**500 ppb CrPic **	1976.50^de^	1400.50^e^	3111.40^bc^	2.23^b^	203.70^e^	45.00^c^	2.37^c^
**1000 ppb CrPic **	2134.00abcd	1598.00^bc^	3215.00^ab^	2.01^c^	261.40^bc^	56.64^a^	2.82^a^
**1500 ppb CrPic **	2107.70bcde	1506.30^cd^	3072.70^bc^	2.05^c^	237.30^cd^	49.07^b^	2.58^b^
**500 ppb Nano-CrPic **	2020.20cde	1444.70^de^	3106.10^bc^	2.15^bc^	214.40^de^	46.43^bc^	2.44^bc^
**1000 ppb Nano-CrPic **	2275.30^ab^	1675.30^b^	3189.60^ab^	1.90^d^	279.20^ab^	52.53^a^	2.76^a^
**1500 ppb Nano-CrPic **	2219.10abc	1570.60^c^	3292.10^a^	2.09^bc^	242.20cde	47.70^bc^	2.51^bc^
***p*** **-value**	< 0.001	< 0.0001	< 0.004	< 0.0001	< 0.0001	< 0.0001	< 0.0001
**Significant**	70.28	31.34	45.30	0.044	10.60	1.07	0.05

1 FCR: Feed conversion ratio,

2 EPE: European production efficiency factor,

3 PI: Performance index, and

4 PER: Protein efficiency ratio.

**abcdef:** Different superscript letters indicate significant differences on each column at *p *< 0.05.

Body weight gain, feed conversion ratio (FCR) and European production efficiency factor improved with supplementation of Cr and nano-chromium in heat-stressed chickens. Supplementation of CrPic at level of 1500 ppb and nano-chromium at 1000 ppb had the best performance. 

The ELISA titers of IB at 21, 28, 35 and 42 days of age are shown in Table 3. The hemagglutination titers of AI at 21, 28, 35 and 42 days of age are shown in Table 4. Results showed that supplementation of CrPic and nano-Cr improved ELISA titers of IB and hemagglutination titers of AI in heat-stressed chickens. The CrPic at level of 1500 ppb and nano-Cr at 1000 ppb had the best results.

**Table 3:** Effect of chromium picolinate and chromium nanoparticles supplementation on ELISA titers of infectious bronchitis. Data are presented as mean ± SEM

**Groups**	**21 days**	**28 days**	**35 days**	**42 days**
**Control**	1245.00 ± 204.04	2371.00 ± 1137.96	2399.50 ± 308.90abc	2576.00 ± 708.70^a^
**Heat stress**	1162.50 ± 354.40	1836.00 ± 385.06	1600.00 ± 361.41^e^	1454.00 ± 216.50^b^
**500 ppb CrPic **	1437.50 ± 337.20	1950.30 ± 376.85	1808.00 ± 251.47^de^	1500.00 ± 457.10^b^
**1000 ppb CrPic **	1275.00 ± 64.59	2187.80 ± 1414.70	2338.30 ± 218.65^bc^	2100.00 ± 338.50^a^
**1500 ppb CrPic **	1237.50 ± 75.00	2402.00 ± 2069.07	2885.80 ± 141.79^a^	2328.00 ± 680.20^a^
**500 ppb Nano-CrPic **	1362.00 ± 375.70	2071.00 ± 1314.02	2446.00 ± 179.44^cd^	2279.00 ± 480.00^a^
**1000 ppb Nano-CrPic **	1417.50 ± 56.80	2619.00 ± 1136.29	2899.30 ± 463.37^a^	2823.00 ± 313.70^a^
**1500 ppb Nano-CrPic **	1335.00 ± 50.66	2159.00 ± 705.41	2771.50 ± 397.31^ab^	2331.80 ± 669.00^a^
**Significant**	NS	NS		

**abcde:** Different superscript letters indicate significant differences on each column at* p *< 0.05; NS: Non-significant,

* : Significant.

**Table 4:** Effect of chromium picolinate and chromium nanoparticles supplementation on hemagglutination titers of avian influenza. Data are presented as mean ± SEM

**Groups**	**21 days**	**28 days**	**35 days**	**42 days**
**Control**	4.50 ± 0.00	3.87 ± 0.25^ab^	3.26 ± 0.63^ab^	3.50 ± 0.40^a^
**Heat stress**	4.00 ± 0.00	2.75 ± 1.68^b^	3.37 ± 1.31^b^	1.75 ± 0.64^b^
**500 ppb CrPic **	3.87 ± 0.25	5.00 ± 1.68^a^	3.37 ± 0.85^ab^	2.37 ± 0.48^b^
**1000 ppb CrPic **	4.12 ± 0.75	5.12 ± 1.65^a^	3.75 ± 0.95^ab^	3.62 ± 0.85^a^
**1500 ppb CrPic **	4.25 ± 0.28	5.12 ± 1.65^a^	4.00 ± 1.78 ab	3.63 ± 0.48^a^
**500 ppb Nano-CrPic **	3.87 ± 0.75	3.37 ± 0.48^ab^	3.62 ± 2.00^ab^	3.50 ± 1.47^a^
**1000 ppb Nano-CrPic **	4.37 ± 0.25	4.75 ± 0.95^a^	4.85 ± 1.70^a^	3.50 ± 0.57^a^
**1500 ppb Nano-CrPic **	4.25 ± 0.86	3.75 ± 0.64^ab^	4.25 ± 0.64^ab^	3.62 ± 0.75^a^
**Significant**	NS			

**abcde:** Different superscript letters indicate significant differences on each column at* p *< 0.05; NS: Non-significant,

* : Significant.

## Discussion

This study was conducted to investigate the effects of different levels CrPic and Nano-CrPic on performance and antibody titers against IB and AI of broiler chickens under heat stress condition. Supplementation of CrPic has beneficial effects on performance and blood biochemical parameters of broiler chickens reared under heat stress conditions.^20^ The CrPic supplementation attenuated the decline in performance and antioxidant status resulting from heat stress in quails.^21^ Increases in lymphocyte counts and decreases in heterophil to lymphocyte ratios in 500 and 3000 ppb Nano-CrPic supplementation in heat-stressed chicks has been reported previously and it has been suggested that Nano-CrPic supplementation exhibits an anti-stress function.^22^

The supplementation of CrPic and nano-Cr could improve body weight gain and feed conservation rate of broilers, these results are in general agreement with previous reports.^17^^,^^23^^,^^24^ Nano-chromium supplementation at level of 1000 ppb improved European production efficiency factor. It has been reported that dietary supplementation of organic chromium does not improve the production performance of broiler chickens.^25^ According to previous studies, chromium yeast supplementation to broiler chickens significantly increased protein percentage in the breast and thigh.^26^ Also, a possibility of better broiler performance through potassium chloride supplementation under conditions of severe heat stress (35 to 38 ^°^C) has been demonstrated.^27^

Vitamins E and minerals such as Cr cause reduction in the serum level of malondialdehyde, the end product of lipid peroxidation, in laying hens.^28^ Disease and stress increase urinary excretion of Cr.^29^ Thus, stress-associated immunosuppression alleviation may be one of the mechanisms by which Cr acts.^30^ Results of this study showed that supplementation of Cr can improve hemagglutination titers of AI. These findings are in agreement with the previous reports indicating that supplementation of chromium chloride improves antibody titers of AI.^31^ Supplemental chromium methionine modulated immuno-suppressive effects of heat stress on cellular and humoral immune responses leading to proportions of heterophils and cytotoxic T lymphocytes reduction.^32^

The CrPic supplementation through feed or drinking water enhances the immune responses by up-regulating interferon-gamma expression after vaccination of New-castle disease in broiler chickens.^33^ Heat stress in broilers causes decreases in CD4^+^ and CD8^+^ lymphocytes and antibody production against sheep red blood cells (SRBC).^34^ Supplementation of nano-Cr improved performance under heat stress condition, but addition of nano-Cr at 1500 ppb did not improve the performance index.

In conclusion, the results of this study indicated that supplementation of Cr and nano-chromium improves performance index and antibody titers against AI and IB under heat stress conditions in broiler chickens.
